# Excitation Energy‐Transfer Processes in the Sensitization Luminescence of Europium in a Highly Luminescent Complex

**DOI:** 10.1002/open.201900012

**Published:** 2019-03-28

**Authors:** Yan‐Jie Huang, Can Ke, Li‐Min Fu, Yu Li, Shu‐Feng Wang, Ying‐Chao Ma, Jan‐Ping Zhang, Yuan Wang

**Affiliations:** ^1^ Beijing National Laboratory for Molecular Science State Key Laboratory for Structural Chemistry of Unstable and Stable Species College of Chemistry and Molecular Engineering and Academy for Advanced Interdisciplinary Studies Peking University Beijing 100871 China; ^2^ Department of Chemistry Renmin University of China Beijing 100872 China; ^3^ Institute of Modern Optics & State Key Laboratory for Artificial Microstructure and Mesoscopic Physics School of Physics Peking University China

**Keywords:** Europium, energy transfer, sensitization luminescence, singlet pathway, time-resolved spectroscopy

## Abstract

The excitation energy transfer (EET) pathways in the sensitization luminescence of Eu^III^ and the excitation energy migration between the different ligands in [Eu(fod)_3_dpbt] [where fod=6,6,7,7,8,8,8‐heptafluoro‐2,2‐dimethyl‐3,5‐octanedione and dpbt=2‐(*N*,*N*‐diethylanilin‐4‐yl)‐4,6‐*bis*(3,5‐dimethylpyrazol‐1‐yl)‐1,3,5‐triazine], exhibiting well‐separated fluorescence excitation and phosphorescence bands of the different ligands, were investigated by using time‐resolved luminescence spectroscopy for the first time. The data clearly revealed that upon the excitation of dpbt, the sensitization luminescence of Eu^III^ in [Eu(fod)_3_dpbt] was dominated by the singlet EET pathway, whereas the triplet EET pathway involving T_1_(dpbt) was inefficient. The energy migration from T_1_(dpbt) to T_1_(fod) in [Eu(fod)_3_dpbt] was not observed. Moreover, upon the excitation of fod, a singlet EET pathway for the sensitization of Eu^III^ luminescence, including the energy migration from S_1_(fod) to S_1_(dpbt) was revealed, in addition to the triplet EET pathway involving T_1_(fod). Under the excitation of dpbt at 410 nm, [Eu(fod)_3_dpbt] exhibited an absolute quantum yield for Eu^III^ luminescence of 0.59 at 298 K. This work provides a solid and elegant example for the concept that singlet EET pathway could dominate the sensitization luminescence of Eu^III^ in some complexes.

## 1 Introduction

The sensitization luminescence of lanthanide ions by chromophores has been widely applied in developing various functional systems such as luminescent probes for medical diagnosis, phosphors of LEDs, photochemical supramolecular devices, light conversion agents of solar cells or plant growth regulation, and luminescent thermometers.^1^, ^2^, ^3^, ^4^, ^5^, ^6^, ^7^, ^8^ The necessity of a chromophore to transfer absorbed light energy to the luminescent states of lanthanide ions is due to the forbidden characteristic of 4f–4f electronic transitions. The sensitization mechanism or excitation energy transfer (EET)pathway of lanthanide luminescence complexes is a fundamental issue dealing with the reasonable design and understanding of antenna‐ligand molecular structures for the various applications.

In most investigated lanthanide complexes, the triplet states of antenna ligands dominate the excitation energy transfer in the sensitization luminescence of lanthanide ions (triplet pathway).^9^, ^10^, ^11^, ^12^, ^13^, ^14^ while the direct experimental evidence for the singlet EET pathway in the lanthanide sensitization luminescence (singlet pathway), which does not involve the triplet states of antenna ligands, had been very limited, although it was proposed by Kleinerman^15^ in 1966 and supported by some indirect evidences.^16^,^17^ The singlet energy‐transfer pathway was first demonstrated in our groups by excited‐state dynamics experiments of a Eu^III^ complex [Eu(tta)_3_dpbt] (dpbt, 2‐(*N*,*N*‐diethylanilin‐4‐yl)‐4,6‐*bis*(3,5‐dimethylpyrazol‐1‐yl)‐1,3,5‐triazine; tta, thenoyltrifluoroacetonate), in which the excitation state of S_1_(dpbt) exhibits an obvious feature of charge transfer.^18^,^19^ After that more europium luminescent complexes capable of being sensitized by singlet intra‐ligand charge transfer states were reported.^20^, ^21^, ^22^ Due to the avoidance of energetic constraint from the dpbt T_1_ state, the singlet pathway endows [Eu(tta)_3_dpbt] and its analog [Eu(tta)_3_bpt] (bpt, 2‐(*N*,*N*‐diethylanilin‐4‐yl)‐4,6‐*bis*(pyrazol‐1‐yl)‐1,3,5‐triazine) with efficient Eu^III^ luminescence under one‐photo excitation in visible region or two‐photon excitation in near‐infrared (NIR) region,^19^,^23^,^24^ which has been applied in studying the tumor‐targeting dynamics of nanocarriers in living animals using a home‐built two‐photon excitation time‐resolved imaging system.^3^

Our previously reported experimental evidences for the singlet pathway involved the well matched lifetime of the singlet state of dpbt in [Eu(tta)_3_dpbt] at 430 nm (1.3 ns) and the rise time constant of ^5^D_1_(Eu^III^) emission at 585 nm (1.8 ns), as well as a large difference between the lifetimes of ^5^D_0_(Eu^III^) (0.48 ms) and T_1_(dpbt) state (3.9 s).^18^ It should be mentioned that in the observed singlet pathway, some intermediate states such as a ligand‐to‐metal charge transfer singlet state (LMCT)^25^ may play an important role in the dpbt‐to‐Eu^III^ energy transfer processes, but T_1_(dpbt) is not involved. A further investigation on the energy migration processes among the excitation states of different ligands in a dpbt‐sensitized Eu^III^ luminescence complex is helpful to demonstrate the dominant role of the singlet EET pathway.^8^ However, the experimental analysis on such energy migration processes has not been reported yet.

**Scheme 1:**
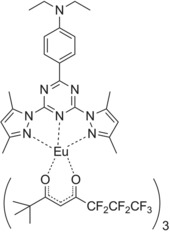
Chemical structure of [Eu(fod)_3_dpbt].

**Figure 1:**
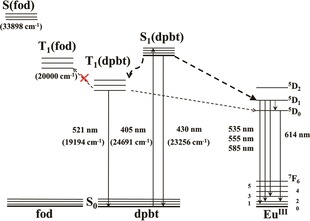
The energy‐transfer pathway in the dpbt‐sensitized luminescence of Eu^III^ in [Eu(fod)_3_dpbt].

In this work, the energy migration processes between the different ligands and the contribution of singlet EET pathways to Eu^III^ luminescence in a highly luminescent complex [Eu(fod)_3_dpbt] (Scheme 1, fod, 6,6,7,7,8,8,8‐heptafluoro‐ 2,2‐dimethyl‐3,5‐octanedione), in which dpbt and fod exhibit well separated fluorescence excitation and phosphorescence bands, were investigated by time‐resolved luminescence spectroscopy and excitation state dynamics analysis. It was demonstrated that upon the excitation of dpbt in [Eu(fod)_3_dpbt], the singlet pathway dominated the sensitization of Eu^III^ luminescence, while the energy migration from T_1_(dpbt) to T_1_(fod) was inactive and the EET pathway of T_1_(dpbt)→Eu^III^ luminescence states was inefficient. (Figure 1). In addition, it is interesting to find that the singlet EET pathway also existed upon the excitation of fod in [Eu(fod)_3_dpbt] besides the triplet EET pathway involving T_1_(fod).

## 2 Results and Discussion

### 2.1 Luminescence Properties and the Singlet Pathway in [Eu(fod)_3_dpbt]

**Figure 2:**
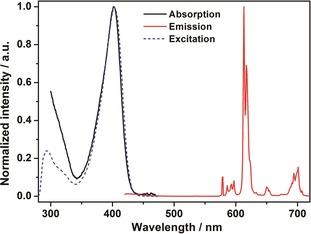
The normalized UV/Vis absorption spectrum (black solid line), Eu^III^ luminescence excitation spectrum (blue dashed line, *λ*_em_=614 nm) and emission spectrum (red solid line, *λ*_ex_=405 nm) of [Eu(fod)_3_dpbt] in toluene (1×10^−5^ m) at 298 K.

Figure 2 shows the normalized UV‐vis absorption, Eu^III^ luminescence excitation and emission spectra of [Eu(fod)_3_dpbt] at 298 K. The two excitation bands at 295 and 405 nm can be attributed to the sensitization function of fod and dpbt, respectively. The absorption band at 405 nm of coordinated dpbt is in consistent with the Eu^III^ luminescence excitation spectrum in the visible light region, while the absorption signal below 350 nm is derived from the absorption band tail of fod. The absolute quantum yield for Eu^III^ luminescence in [Eu(fod)_3_dpbt] upon excitation at 410 nm at 298 K was measured to be 0.59, which was much higher than that of [Eu(tta)_3_dpbt] (0.45). The lifetime of ^5^D_0_(Eu^III^) state in [Eu(fod)_3_dpbt], emitting at 614 nm, was measured to be 684 μs at 298 K, which was longer than that in [Eu(tta)_3_dpbt] (480 μs), implying more efficient depression of the nonradiative deactivation of Eu^III^ luminescent states by the replacement of tta with fod in the complex.

**Figure 3:**
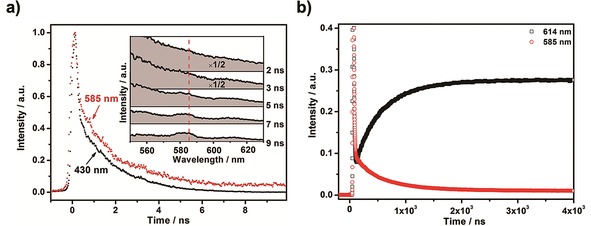
The time‐resolved luminescence spectra and kinetics curves on different timescales of [Eu(fod)_3_dpbt] in toluene recorded at 298 K (1×10^−4^ m). a) Kinetics curves at 430 and 585 nm (*λ*_ex_ = 400 nm), and the inset shows the luminescence spectra at different delay times. b) Kinetics curves at 585 and 614 nm (*λ*_ex_=416 nm).

As shown in Figure 3, when dpbt in [Eu(fod)_3_dpbt] was excited at 298 K, the fluorescence of dpbt at 430 nm exhibited a decay time constant of 1.7 ns that was very close to the luminescence rise time constant (1.8 ns) of Eu^III^ at 585 nm derived from ^5^D_1_(Eu^III^)→^7^F_3_(Eu^III^) transition (see Figure S1 for the fitting results of the luminescence kinetic curves at 430 and 585 nm of [Eu(fod)_3_dpbt]). Moreover, the luminescence decay time constant (Figure 3b) of Eu^III^ at 585 nm was 604 ns which was in the same level with the luminescence rise‐time constant of Eu^III^ at 614 nm (582 ns, corresponding to ^5^D_0_(Eu^III^)→^7^F_2_(Eu^III^) transition).

The inset of Figure 3a shows the dynamic luminescence spectra of [Eu(fod)_3_dpbt] within 10 ns. With the delay time increasing, the background of the fluorescence of dpbt in the complex gradually decreased and the luminescence signal at 585 nm of Eu^III^ luminescence emerged (red dash line). Note that the luminescence rise‐time constant of Eu^III^ luminescence at 614 nm was 582 ns, and the ^5^D_0_(Eu^III^) state had not been adequately populated within 10 ns, so the luminescence signal of Eu^III^ luminescence at 614 nm in the illustration of Figure 3a was not obvious.

The aforementioned tight decay‐to‐rise correlation clearly demonstrated the singlet EET pathway in the dpbt‐sensitized Eu^III^ luminescence of [Eu(fod)_3_dpbt].

### 2.2 Time‐Resolved Phosphorescence Spectroscopy of [Eu(fod)_3_dpbt]

**Figure 4:**
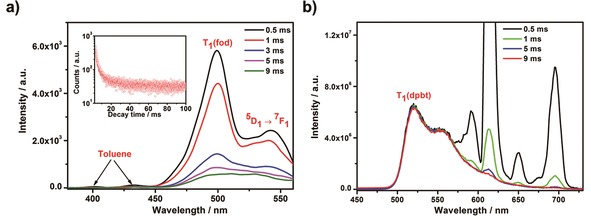
Phosphorescence spectra of [Eu(fod)_3_dpbt] in toluene (5×10^−4^ m) at 77 K with different delay times. a) *λ*_ex_=295 nm, the inset shows phosphorescence intensity decay curve at 500 nm; b) *λ*_ex_=416 nm.

The phosphorescence spectra of dpbt and fod in [Eu(fod)_3_dpbt] at 77 K measured 0.5 ms after the photoexcitation of dpbt and fod, respectively, are shown in Figure 4. When fod in [Eu(fod)_3_dpbt] was excited at 295 nm (Figure 4a), besides the phosphorescence bands at 400, 428 nm of toluene,^26^,^27^ and the peak at 540 nm of ^5^D_1_→^7^F_1_ transition of Eu^III^, the phosphorescence band of T_1_(fod)→S_0_(fod) at 500 nm (20000 cm^−1^) was observed clearly. The phosphorescence decay at 500 nm could be well fitted by a bi‐exponential function with the time constants or lifetimes of 2.1 ms (39.3 %) and 16.5 ms (60.7 %), suggesting that there exist two kind of T_1_(fod) with different coordination environments in the compound. Upon the excitation at 295 nm at 77 K, the Eu^III^ luminescence decay curve at 614 nm (Figure S2a) could be well fitted by a bi‐exponential function with the lifetimes of 826 μs (93.1 %) and 2.1 ms (6.9 %). The long lifetime of ^5^D_0_(Eu^III^) was well matching with the short lifetime of T_1_(fod) (2.1 ms), suggesting the existence of EET from T_1_(fod) to the Eu^III^ luminescence states upon the excitation of fod, while the short lifetime of ^5^D_0_(Eu^III^) was the characteristic decay constant for the deactivation process of Eu^III^ in [Eu(fod)_3_dpbt]. Moreover, the shape of the phosphorescence spectrum at 500 nm changed with delay time increasing, which may be induced by the presence of phosphorescence of the long‐lifetime T_1_(fod) and T_1_(dpbt).

When dpbt in [Eu(fod)_3_dpbt] was excited at 416 nm (Figure 4b), the phosphorescence spectrum corresponding to T_1_(dpbt)→S_0_(dpbt) transition at 77 K exhibited a broad band with a maximum wavelength of 521 nm (19194 cm^−1^), and the decay curve of the phosphorescence intensity could be well fitted with a single exponential function with a time constant or lifetime of 3.3 s (Figure S3). The narrow emission bands at 591, 614, 650, 695 nm could be attributed to the Eu^III^ luminescence signals. The phosphorescence signal of the ligand fod (at 500 nm) could not be observed in the phosphorescence spectrum of [Eu(fod)_3_dpbt] upon the excitation at 416 nm, which was in consistent with the fact that the energy level of T_1_(fod) was higher than that of T_1_(dpbt) by 806 cm^−1^, indicating that the energy migration process T_1_(dpbt)→T_1_(fod) hardly happened in [Eu(fod)_3_dpbt].

During the period from 0.5 ms to 9 ms after the photoexcitation, the luminescent intensity of ^5^D_0_(Eu^III^)→^7^F_4_(Eu^III^) transition at 695 nm decreased by 98.8 %, whereas the phosphorescence intensity of T_1_(dpbt)→S_0_ at 521 nm was nearly unchanged, indicating that T_1_(dpbt) was not the main energy donor for the sensitization of Eu^III^ luminescence in [Eu(fod)_3_dpbt], and any energy transfer process started from T_1_(dpbt) was inefficient within 9 ms.

A weak emission band at 614 nm of ^5^D_0_(Eu^III^)→^7^F_2_ (Eu^III^) was observed in the phosphorescence spectrum of [Eu(fod)_3_dpbt] measured 9 ms after the pulse excitation of dpbt at 416 nm. Since the Eu^III^ luminescence at 614 nm derived from the singlet‐pathway sensitization has a lifetime of 963 μs as measured at 77 K (Figure S2b), its intensity would decay at least by one ten thousandth after 9 ms. The Eu^III^ luminescence at 614 nm derived from the triplet‐pathway sensitization should have an apparent lifetime of 3.3 s (the same as that of T_1_(dpbt)) at 77 K, and its intensity would not decrease obviously within 9 ms. Therefore, the contribution to Eu^III^ luminescence from the triplet energy transfer pathway could be roughly estimated to be less than 2 % (see supporting information), implying that the population of T_1_(dpbt) state is inefficient for loosening the forbidden rule of f‐f transition of Eu^III^ in [Eu(fod)_3_dpbt]. It should be mentioned that, compared with the case at room temperature, the much longer lifetime of T_1_(dpbt) at 77 K enhanced the contribution of triplet EET pathway to the sensitization luminescence of Eu^III^.

**Figure 5:**
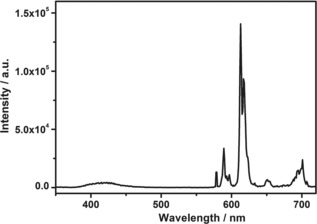
Luminescence spectrum of [Eu(fod)_3_dpbt] in toluene (1×10^−5^ m) at 298 K under the excitation at 295 nm.

Furthermore, it was found that when fod in [Eu(fod)_3_dpbt] was excited at 298 K, the fluorescence of dpbt at 430 nm appeared clearly (Figure 5), which may be derived from the Förster resonance energy transfer from fod to dpbt, implying that the singlet EET process for the sensitized luminescence of Eu^III^ in [Eu(fod)_3_dpbt] also existed upon the excitation of fod and excitation energy migration. Such a unique sensitization manner and the excellent luminescent properties make [Eu(fod)_3_dpbt] to be a promising luminescence dye in the analysis based on photoinduced luminescence.^28^,^29^

## 3 Conclusions

In conclusion, the energy migration processes between the different ligands and the EET pathways for Eu^III^ sensitization luminescence in a highly luminescent complex [Eu(fod)_3_dpbt] were investigated. The obtained data including the well matched fluorescence lifetime of dpbt at 430 nm and the luminescence rising time constant of ^5^D_1_(Eu^III^)→^7^F_3_(Eu^III^) transition at 585 nm, the relationships among the lifetimes of T_1_(dpbt), T_1_(fod), ^5^D_0_(Eu^III^), as well as the phosphorescence spectra measured with different delay times clearly revealed that the singlet EET pathway dominate the sensitization luminescence of Eu^III^ contribution of more than 98 % at 77 K upon the excitation of dpbt. It was found that the energy migration from T_1_(dpbt) to T_1_(fod) hardly happened in [Eu(fod)_3_dpbt] under excitation of dpbt, while upon the excitation of fod, there was a singlet EET process for the sensitization of Eu^III^ luminescence including the energy migration from S_1_(fod) to S_1_(dpbt), in addition to the triplet EET pathway.

## **Experimental Section**

The dpbt ligand and the [Eu(fod)_3_dpbt] complex were synthesized according to the previously reported methods.^18^,^30^ In a typical experiment for preparing [Eu(fod)_3_dpbt], a solution of Eu(fod)_3_⋅3H_2_O (103.8 mg, 0.1 mm) in benzene (10 mL) was added to a solution of dpbt (41.6 mg, 0.1 mm) in benzene (10 mL) under argon, and the mixture was stirred for 3 h to give a yellow solution with bright red luminescence in daylight. ^1^H NMR (C_6_D_6_, 400 MHz, 298 K, TMS): *δ*=30.553 (s, 6H; Pz‐CH_3_), 13.073 (s, 2H; Pz−H), 7.557 (d, ^3^J(H,H)=9.0 Hz, 2H; Ph−H), 6.059 (d, ^3^J(H,H)=8.9 Hz, 2H; Ph−H), 4.673 (s, 6H; Pz‐CH_3_), 2.687 (q, ^3^J(H,H)=7.4 Hz, 4H; NCH_2_CH_3_), 0.636 (t, ^3^J(H,H)=7.0 Hz, 6H; NCH_2_CH_3_) 0.153 (s, 27 H; C‐CH_3_), −1.567 (s, 3H; CH) ppm. EI MS: m/z: 1157.3 ([M‐fod]^+^). Elemental analysis (EuC_53_H_58_N_8_F_21_O_6_): found C 43.55, H 3.97, N 7.70; calc. C 43.78, H 4.02, N 7.71.

The steady state photoluminescence spectra of the complexes were measured on a fluorescence lifetime and steady state spectrometer (FLS 980, Edinburgh Instruments) equipped with an Oxford Optistat DN2 cryostat and a PMT detector. In the phosphorescence spectra measurements on FLS 980, the delay time (time interval between the excitation pulse and spectrum measurement) could be adjusted in a range from 5 μs to 9.0 ms with an exposure time of 1 ms. A quartz cuvette with an optical path length of 1 cm was used in the measurements.

Absolute quantum yields of [Eu(fod)_3_dpbt] were measured on an Hamamatsu Photonics C9920‐02 absolute quantum yield spectrometer, which is more accurate than the quantum yields measured by relative method. UV‐Vis absorption spectrum was recorded on a UV3600 PLUS spectrophotometer in a 1 cm quartz cell. The luminescence lifetimes at microsecond and second timescale were measured on a Horiba Jobinyvon Deltaflex UltraFast lifetime Spectrofluorometer.

The kinetics curves of the Eu^III^ luminescence at 585 nm and the fluorescence of dpbt at 430 nm in [Eu(fod)_3_dpbt] upon the excitation at 400 nm were recorded with a high‐resolution streak camera system (Hamamatsu C10910). We used an amplified mode‐lock Ti: Sapphire femtosecond laser system (Legend, Coherent) to generate the pump beam with a repetition rate of 1 KHz. The samples were excited by the 400 nm laser, which was generated by the 800 nm pulsed laser passing through a frequency‐doubling BBO crystal with an incident angle of ca. 45°.

The luminescence lifetime of μs timescale (Figure 3b) was measured on a digital oscilloscope (bandwidth 600 MHz; LeCroy WaveSurfer 64Xs, Chestnut Ridge). The excitation pulses (0.25 mJ/pulse for 416 nm, 7 ns, 10 Hz) were obtained with a hydrogen‐charged Raman shifter (4 MPa) which was pumped by the third harmonics of a Nd:YAG laser (355 nm, 10 Hz, 70 mJ/pulse, Quanta‐Ray Pro‐Series, Spectra Physics Lasers Inc.). The luminescent light was sent to a spectrograph (SP2500i, Princeton Instruments, USA), and the signal output was fed to the digital oscilloscope.

The time evolution profiles at selected wavelengths were fitted to single or multiple exponential decay functions to derive the decay time constants or lifetimes. The kinetics data was fitted by mathematical functions with Origin 8.5 and Matlab 5.2.

## Conflict of interest

The authors declare no conflict of interest.

## Supporting information

As a service to our authors and readers, this journal provides supporting information supplied by the authors. Such materials are peer reviewed and may be re‐organized for online delivery, but are not copy‐edited or typeset. Technical support issues arising from supporting information (other than missing files) should be addressed to the authors.

Supplementary
