# Supplementary material for: Excitation Energy‐Transfer Processes in the Sensitization Luminescence of Europium in a Highly Luminescent Complex
Source: ChemistryOpen. 2019 Mar 28;8(3):388–92. doi: 10.1002/open.201900012 (PMC6438127; doi:10.1002/open.201900012)
Supplement: Supplementary file 1 — Supplementary [file OPEN-8-388-s001.pdf]

Supporting Information

© Copyright Wiley-VCH Verlag GmbH & Co. KGaA, 69451 Weinheim, 2019

Excitation Energy-Transfer Processes in the Sensitization Luminescence of Europium in a Highly Luminescent Complex

Yan-Jie Huang[+], Can Ke[+], Li-Min Fu, Yu Li, Shu-Feng Wang, Ying-Chao Ma, Jan-Ping Zhang,* and Yuan Wang*© 2019 The Authors. Published by Wiley-VCH Verlag GmbH & Co. KGaA. This is an open access article under the terms of the Creative Commons Attribution License, which permits use, distribution and reproduction in any medium, provided the original work is properly cited.

Supporting Information

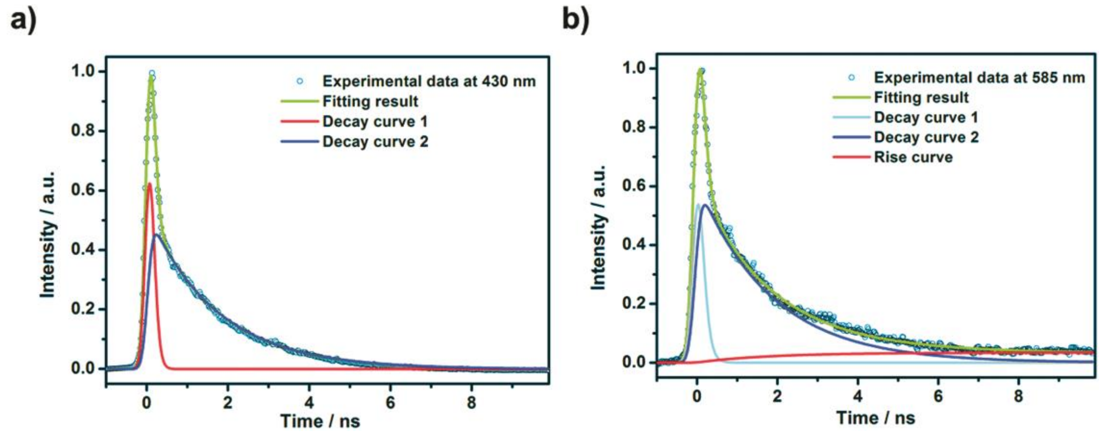

Figure S1. The fitting results of luminescence kinetics curves at 430 (a) and 585 nm (b) of [Eu(fod)$_3$dpbt] in toluene (5 $\times 10^{-4}$ M) at 298 K, λ_{ex} = 400 nm.

As shown in Figure S1a, the luminescence decay kinetic curve at 430 nm of dpbt in [Eu(fod)$_3$dpbt] could be well fitted by a bi-exponential function with the lifetimes of 0.06 $\pm$ 0.002 ns (pre-exponential factor: 2.92) and 1.7 $\pm$ 0.008 ns (pre-exponential factor: 0.53). It should be mentioned that the plus/minus values of time constant were given by data fitting, while at present test setting (streak camera time window was 10 ns), the error caused by the measurement method would be no more than 0.1 ns.

The decay curve 1 (red line) is the fitting result faster than the temporal resolution of the streak camera in the current time interval.[1] The decay curve 2 (blue line) shows the fluorescence decay kinetics of dpbt in [Eu(fod)$_3$dpbt], corresponding to the transition from S_1(dpbt) to S_0(dpbt). Figure S1b shows the fitting results of the luminescence kinetics at 585 nm of [Eu(fod)$_3$dpbt]. The lifetimes of the decay curve 1 and 2 in Figure S1b are 0.10 $\pm$ 0.005 ns (pre-exponential factor: 2.01) and 1.8 $\pm$ 0.014 ns (pre-exponential factor: 0.63), respectively, which are close to those in Figure S1a, i.e. within 10 ns the decaying signal at 585 nm is mainly derived from the tail of fluorescence band at 430 nm. Besides of these two decay components, a luminescence intensity rise curve at 585 nm with a time constant of 1.8 $\pm$ 0.014 ns (pre-exponential

factor: 0.034) can be clearly seen in Figure S1b, corresponding to the electron population of $^5D_1$ level in [Eu(fod)$_3$dpbt]. It should be mentioned that although the decay curve 1 has a large contribution in the fitting results, its lifetime is so short (0.10 ns) that curve 1 has little effect on the fitting result after 1 ns. These tight decay-to-rise correlations clearly revealed the singlet EET pathway in the dpbt-sensitized Eu^{III} luminescence of [Eu(fod)$_3$dpbt].

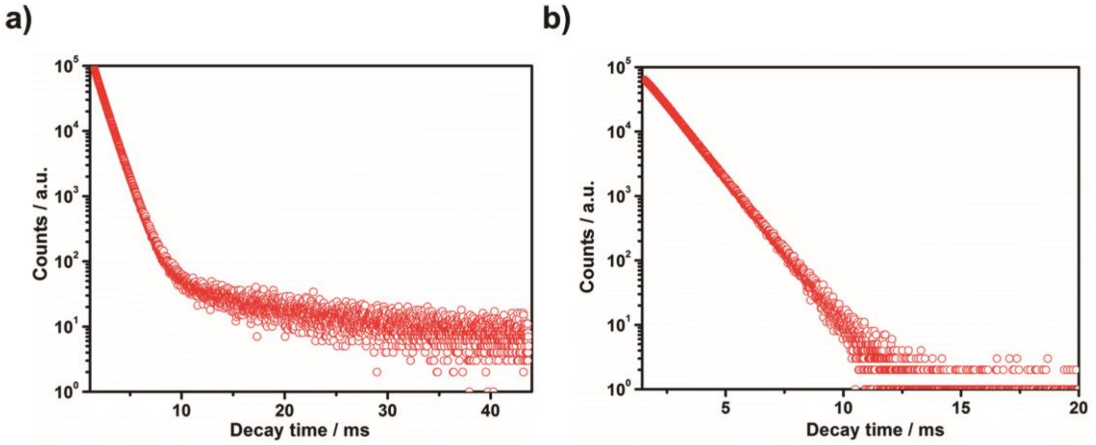

Figure S2. The phosphorescence decay kinetics of Eu^{III} luminescence at 614 nm of [Eu(fod)$_3$dpbt] in toluene (5 $\times 10^{-4}$ M) at 77 K. (a) λ_{ex} = 295 nm; (b) λ_{ex} = 416 nm.

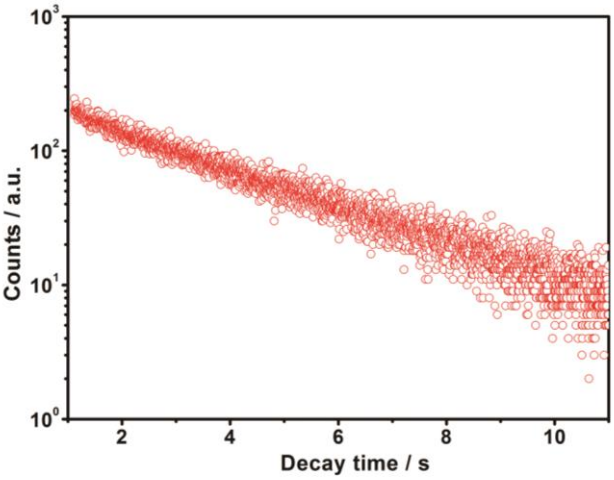

Figure S3. The phosphorescence decay kinetics of dpbt at 521 nm of [Eu(fod)$_3$dpbt] in toluene (5 $\times 10^{-4}$ M) at 77 K, λ_{ex} = 416 nm.

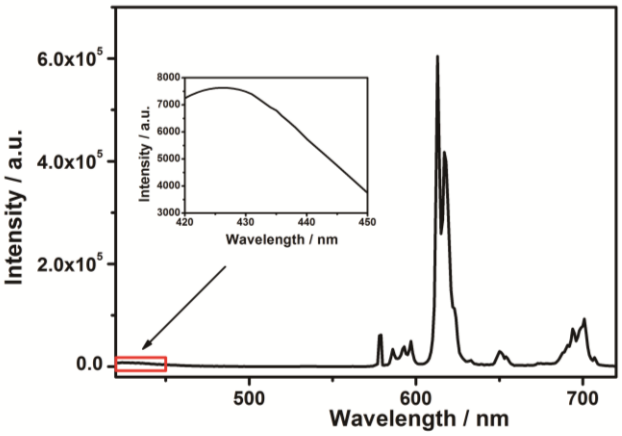

Figure S4. Luminescence emission spectrum of [Eu(fod)$_3$dpbt] in toluene at 298 K (1 ×10^{-5} M) under the excitation at 405 nm. The insert is an enlarged spectrum between 420 and 450 nm.

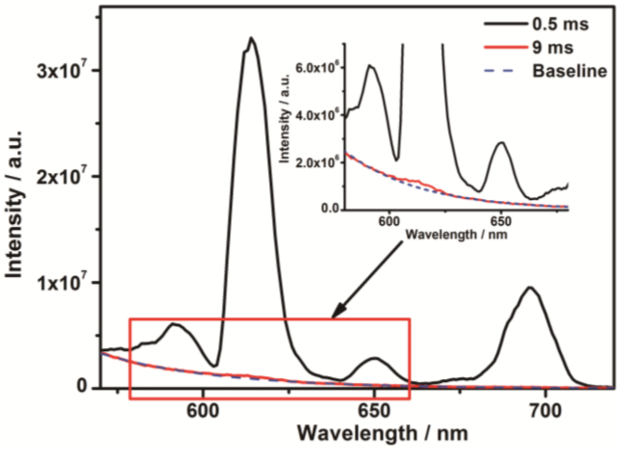

Figure S5. Phosphorescence spectra of [Eu(fod)$_3$dpbt] in toluene at 77 K (5 ×10^{-4} M) measured with a delay time of 0.5 ms (black line) and 9 ms (red line) after the pulse excitation at 416 nm, respectively. The blue dotted line was the baseline and the inset shows the amplified spectra in the range from 580 to 680 nm.

A weak emission band at 614 nm of $^5D_0(Eu^{III}) \rightarrow {}^7F_2$ (Eu^{III}) was observed in the phosphorescence spectra of [Eu(fod)$_3$dpbt] measured 9 ms after the pulse excitation of dpbt at 416 nm, which contains the contribution from the triplet pathway to the sensitization of Eu^{III} luminescence. Therefore, the corresponding Eu^{III} luminescence intensity derived from triplet EET pathway is less than or close to the integral value of

the red curve (Figure S5) subtracting that of the baseline in the range from 604 to 639 nm (4.1×10^6), because the Eu^{III} luminescence at 614 nm derived from the triplet-pathway sensitization should have an apparent lifetime of 3.3 s (the same as that of T_1(dpbt)) at 77 K, and its intensity would not decrease obviously within 9 ms. In this experiment, [Eu(fod)$_3$dpbt] was excited by a light pulse produced by a μs -lamp of FLS 980 every 10 ms and the signal measurement time was 0.5 s, resulting in the accumulation of the signal for 50 times. Since the lifetime of the Eu^{III} luminescence at 614 nm derived from triplet EET pathway was about 3.3 s, this phosphorescence signal with long lifetime was repeatedly accumulated many times which could be estimated to be:

$$0+1+2+\cdots+49 = 1225$$

Then the real luminescence intensity derived from triplet EET pathway could be roughly estimated as:

$$4.1\times10^6 / 1225 = 3.3\times10^3$$

On the other hand, the corresponding Eu^{III} luminescence intensity derived from singlet EET pathway must be larger than the integral value of the black curve (Figure S5) subtracting that of the baseline in the range from 604 to 639 nm (4.2×10^8).

The luminescence intensity at any time can be expressed as:

$$I_t = I_0 e^{-t/\tau}$$

Where I_0 is the initial intensity, thus $I_{0.5ms} = I_0 e^{-0.5/0.963}$, and I_0 derived from the singlet EET pathway was estimated to be $4.2\times10^8\times1.7 = 7.1\times10^8$. Moreover, the integral area of a single-exponential decay curve is calculated to be $I_0\tau$. Therefore, the contribution to the dpbt-sensitized Eu^{III} luminescence of $^5D_0(Eu^{III}) \rightarrow {}^7F_2(Eu^{III})$ transition from the triplet EET pathway in [Eu(fod)$_3$dpbt] could be roughly estimated by these values :

$$(3.3\times10^{3}\times3200) / (3.3\times10^{3}\times3200 + 7.1\times10^{8}\times0.963) = 1.5\%$$

[1] L. M. Fu, X. C. Ai, M. Y. Li, X. F. Wen, R. Hao, Y. S. Wu, Y. Wang, J. P. Zhang, *J. Phys. Chem. A.* **2010**, *114*, 4494-4500.
